# Gastrointestinal stromal tumor of the small intestine presenting as a giant intratumoral abscess: a rare case report

**DOI:** 10.1093/jscr/rjaf344

**Published:** 2025-05-31

**Authors:** Saleh Husam Aldeligan, Abdullah Aljunaydil, Nuha Qattan, Ashwaq A Almajed, Hassan Hifni, Fahad Bamehriz

**Affiliations:** College of Medicine, King Saud University, PO Box 2925, Riyadh 11461, Saudi Arabia; Department of Surgery, King Saud University Medical City, PO Box 7805 #37, Riyadh 11472, Saudi Arabia; Department of Surgery, King Saud University Medical City, PO Box 7805 #37, Riyadh 11472, Saudi Arabia; Department of Surgery, King Saud University Medical City, PO Box 7805 #37, Riyadh 11472, Saudi Arabia; Department of Surgery, King Saud University Medical City, PO Box 7805 #37, Riyadh 11472, Saudi Arabia; Department of Surgery, King Saud University Medical City, PO Box 7805 #37, Riyadh 11472, Saudi Arabia

**Keywords:** gastrointestinal stromal tumor, small intestine, intratumoral abscess, case report

## Abstract

Gastrointestinal stromal tumors (GIST) are rare, but they are the most common mesenchymal tumors of the gastrointestinal tract. Here, we present a case of 48 years medically free female presenting with progressive abdominal distension associated with abdominal discomfort for 2 months. On physical examination, she was hemodynamically stable with a hugely distended abdomen but soft and lax. Laboratory findings revealed leukocytosis and 16 other labs were unremarkable. Computed tomography (CT) of the abdomen showed a large cystic mass likely arising from small bowel loops measuring 30 × 19 × 22 cm. The lesion has a peripheral soft tissue component measuring 3 cm in thickness enhanced with contrast material, suggesting hypervascularity. A large air-fluid level with a large volume of intra-cystic fluids was present, which is concerning for associated enteric fistula. No bowel obstruction was observed. The plan was to start the patient on antibiotics and to proceed with diagnostic laparoscopy for cyst excision with possible resection and anastomosis of the small bowel. Furthermore, she was discharged on day 5 with an uneventful postoperative course as the patient was started on IV antibiotics postoperatively and was discharged on oral antibiotics to complete 14 days. 6-month follow-up patient started on imatinib. Although GISTs presenting as predominantly cystic lesions are very rare, they should be considered one of the differential diagnoses of such a presentation.

## Introduction

Gastrointestinal stromal tumors (GISTs) are the most common malignant subepithelial lesions of the gastrointestinal tract, with an incidence of 10–15 cases per million and a median age of diagnosis in the mid-60s. They arise from Cajal interstitial cells, driven by mutations in KIT tyrosine kinase or platelet-derived growth factor receptor alpha (PDGF-r-α) oncogenes. GISTs are most frequently found in the stomach (lesser or greater curvature), followed by the small intestine, colon, rectum, and esophagus. While often asymptomatic, they may present with gastrointestinal bleeding, melena, hematemesis, or mass effects. Approximately 15%–30% of GISTs are discovered incidentally during surgery or imaging for other conditions [1–6]. Diagnosis relies on histological examination of biopsies obtained via endoscopic ultrasound-guided fine needle aspiration (EUS-FNA), as computed tomography (CT) and magnetic resonance imaging (MRI) are less effective, and visual endoscopy often misses submucosal tumors. Surgical resection is the primary treatment for localized GISTs, with laparoscopic techniques preferred for small tumors and open laparotomy for larger or complex cases. For metastatic GISTs, tyrosine kinase inhibitors (TKIs) like imatinib are highly effective and often used postoperatively to prevent recurrence [7–9]. GISTs can disrupt the mucosal barrier, leading to bacterial translocation, abscess formation, and bacteremia. Although rare, intratumoral abscesses can occur, particularly in small intestinal GISTs, which are more prone to complications like obstruction, perforation, and abscess formation compared to gastric GISTs. Liver and peritoneal abscesses are more common than intratumoral abscesses, which are exceptionally rare and poorly documented in the literature [10–12]. This report highlights an unusual case of a small intestinal GIST presenting with a giant intratumoral abscess exceeding 25 cm, the largest documented in the literature. While most GIST-associated abscesses range from 5–15 cm, this case underscores the potential for rare, severe complications in small intestinal GISTs, emphasizing the need for prompt diagnosis and management.

## Case presentation

A 48 year old otherwise healthy female came to our emergency room complaining of progressive abdominal distension for 2 months; she had no abdominal pain or discomfort. She had no fever, no vomiting, and she passed bowel motions. Her blood pressure was 132/69 and heart rate was 106 beats/min. Physical examination revealed a severely distended but soft and lax abdomen. Laboratory testing showed elevated WBC (16.4 × 10^9^/L), hemoglobin (94 g/L), and neutrophils ratio (78.6%). A contrast enhanced CT scan of the abdomen and pelvis showed a large cystic mass not clearly from where it was arising but it was most likely arising from small bowel loops measuring 30 × 19 × 22 cm. The lesion has a peripheral soft tissue component measuring 3 cm in thickness enhanced with contrast material, suggesting hypervascularity. A large air-fluid level with a large volume of intra-cystic fluids was present, which is concerning for associated enteric fistula. No bowel obstruction was observed. The plan was to start the patient on antibiotics and to proceed with diagnostic laparoscopy for cyst excision with possible resection and anastomosis of the small bowel. The patient underwent emergency laparoscopic exploration, where she was found to have a large pelviabdominal encapsulated cystic lesion measuring 28 cm extending from the right upper quadrant to the pelvis and crossing the midline, very thick capsule attached inferiorly to the bowel and superiorly to the transverse colon and omentum, thickened fibrotic appendix attached to the sigmoid mesentery at rectosigmoid junction, multiple segments of bowel attached to the inferior surface of the capsule and it was inseparable from the jejunum. The capsule was opened and 4 L of thick foul-smelling pus were aspirated. Dissection of the cyst started from the right upper quadrant freeing the small bowel from the cyst and carried out until the rectosigmoid attachment. Resection of involved segments of the small bowel was done with primary anastomosis, due to the attachment of the appendix to the cyst and the rectosigmoid mesentery an ileocolic resection was carried out. A lower midline incision was done to retrieve the specimen (small bowel, ileocolic, cyst), and three drains were inserted, pelvis, subhepatic, and right paracolic gutter. A tumor sample was sent for cytology, bacterial, fungal, and acid-fast bacilli cultures. Postoperatively the patient started a clear liquid diet and progressed to a normal diet with no nausea or vomiting, drains were kept for five days and removed after. The patient started on IV antibiotics postoperatively and was discharged on oral antibiotics to complete 14 days. She was discharged on day 5 with an uneventful postoperative course. Then the patient was followed for 6 months, and the patient was started on imatinib. The pathology department received a segment of ileocecal and an opened partially fragmented cyst. The ileocecal segment weighs 263 grams and measures 25 × 3 × 2 cm. The cyst weighs 1,070 g and measures 28 × 28 × 4.5 cm. The sample revealed mixed epithelioid and spindle cell type GIST, with prominent cystic changes, extensive exudative ulceration, and necrosis. The mitotic rate was 0–1 mitotic figure/5 mm square. Immunohistochemical staining showed that the tumor was positive for CD117, DOG1, and vimentin, and negative for pan-cytokeratin, desmin, SMA, S100, calretinin, CD68, CD31, and CD34. Ki67 index was 5%. The patient was discharged from the hospital on the fifth postoperative day and has been treated with imatinib in the outpatient clinic without any medical problems.

## Discussion

GISTs are predominantly solid tumors with cystic changes being rare [6]. The most common location is the stomach (60%), followed by jejunum and ileum (30%), duodenum (5%), and colorectum (<5%) [3]. Clinical presentations of gastric and small bowel GIST could be nonspecific complaints such as early satiety and bloating. Occasionally they can ulcerate and bleed or grow large enough to cause pain or obstruction [1]. Symptomatic colorectal GISTs may present with lower GI bleeding, perforation, pain, or obstruction, rarely an externally palpable mass may present with malignant GISTs [13]. In rare cases, GIST can present with severe hypoglycemia due to paraneoplastic production of insulin-like growth factor II [14]. Although GISTs can be implied clinically by the symptoms, however, they can be asymptomatic and discovered incidentally on imaging [11]. It was reported in a previous study that the median tumor size was 7 cm, and only 28% of the tumors were over 10 cm. 5 It is uncommon to find predominant cystic neoplasms in GISTs, as they are typically solid tumors with a small cystic area [6], a few case reports showed submucosal tumors associated with abscess formation. It is very rare to see a case with bacterial-induced abscess in the gastric wall and they usually have a poor prognosis with a high mortality rate because the diagnosis is usually late [11]. The most common pathogens are Streptococci, Staphylococci, *E. coli*, *Haemophilus influenzae*, Proteus, and Clostridium [12]. The predisposing factors to the formation of the localized gastric wall abscess are Alcoholism, diabetes mellitus, decreased gastric acidity, and immunosuppression [11]. GISTs can be detected by endoscopy; however, because of the limitation of endoscopy in tumor diagnosis and assessment we can further assess GISTs by contrast-enhanced CT and/or endoscopic ultrasonography (EUS) but only in case of larger than 2 cm GISTs as an initial workup, if it was smaller than 2 cm with no high-risk features we followed it periodically by EUS [8]. To know the exact location of the tumor, endoscopic ultrasound is the imaging of choice. In case of an anorectal involvement you can use MRI to see more anatomical details. The prognosis of GISTs depends on many factors, such as the mitotic index, tumor size, tumor location (gastric vs. nongastric), type of mutation, tumor rupture, and whether it occurred before or during surgery [8]. The goal in treating GISTs is to improve the symptoms and to eradicate the malignant lesion so the indication to start the patient on treatment is either symptomatic or lesion with malignant features or confirmed in pathology [7]. All GISTs that are larger than 2 cm are considered malignant [7]. GISTs are either localized or advanced, which differs in the management, for localized tumors, the main management is surgical resection. The goal of the surgery is to eradicate the tumor and to avoid the tumor rupture [7]. The surgical resection is either open or laparoscopic but for gastric GISTs that are smaller than 5 cm laparoscopic is safer because it is less invasive with similar oncological outcomes [8]. In the case of advanced tumors, it is either resectable or unresectable; for resectable tumors, surgical resection is the treatment of choice with acceptable morbidity but a high recurrence rate; for unresectable tumors, Imatinib is the treatment of choice [1]. This case stands out due to the exceptionally rare presentation of a jejunal GIST with a giant intratumoral abscess, a complication more commonly reported in gastric GISTs. While a few reports, such as Vico *et al.* [11], describe gastric GISTs complicated by abscess formation, similar findings in the small intestine remain extremely rare. Additionally, Fakhrejahani *et al.* [12] reported a GIST mimicking a liver abscess, underscoring the diagnostic challenge such presentations pose. Compared to those cases, our report adds to the limited body of evidence on jejunal GISTs manifesting with large abscess cavities and illustrates the importance of early recognition and appropriate surgical management to prevent rupture and sepsis. Moreover, our patient’s abscess was confined within the tumor, and the surgical approach prioritized en bloc resection without spillage, which is crucial for prognosis. The rarity of this presentation and the successful surgical outcome despite the risk of rupture underscore the clinical significance of our case.

## Conclusion

An intratumoral abscess of the small intestinal GIST is rare; this case report describes a rare case of a huge GIST in the small intestine. In this case, a large GIST was associated with a closed intratumoral abscess, necessitating emergency surgery.
